# Characterizing influence of rCHOP treatment on diffuse large B-cell lymphoma microenvironment through in vitro microfluidic spheroid model

**DOI:** 10.1038/s41419-023-06299-6

**Published:** 2024-01-09

**Authors:** Matthew R. Sullivan, Rachel P. White, Dashnamoorthy Ravi, Ninad Kanetkar, Ilana Berger Fridman, Adam Ekenseair, Andrew M. Evens, Tania Konry

**Affiliations:** 1 Department of Pharmaceutical Sciences, Northeastern University, Boston, MA USA; 2 Rutgers Cancer Institute, New Brunswick, NJ USA; 3 Chemical Engineering Department, Northeastern University, Boston, MA USA; 4 Avram and Stella Goldstein-Goren Department of Biotechnology and Regenerative Medicine and Stem Cell Center, Ben-Gurion University of the Negev, Beer-Sheva, Israel

**Keywords:** Cancer microenvironment, Drug development, Tumour immunology, B-cell lymphoma, Cell death and immune response

## Abstract

For over two decades, Rituximab and CHOP combination treatment (rCHOP) has remained the standard treatment approach for diffuse large B-cell lymphoma (DLBCL). Despite numerous clinical trials exploring treatment alternatives, few options have shown any promise at further improving patient survival and recovery rates. A wave of new therapeutic approaches have recently been in development with the rise of immunotherapy for cancer, however, the cost of clinical trials is prohibitive of testing all promising approaches. Improved methods of early drug screening are essential for expediting the development of the therapeutic approaches most likely to help patients. Microfluidic devices provide a powerful tool for drug testing with enhanced biological relevance, along with multi-parameter data outputs. Here, we describe a hydrogel spheroid-based microfluidic model for screening lymphoma treatments. We utilized primary patient DLBCL cells in combination with NK cells and rCHOP treatment to determine the biological relevance of this approach. We observed cellular viability in response to treatment, rheological properties, and cell surface marker expression levels correlated well with expected in vivo characteristics. In addition, we explored secretory and transcriptomic changes in response to treatment. Our results showed complex changes in phenotype and transcriptomic response to treatment stimuli, including numerous metabolic and immunogenic changes. These findings support this model as an optimal platform for the comparative screening of novel treatments.

## Introduction

Diffuse large B-cell lymphoma (DLBCL) is the most common form of non-Hodgkin’s lymphoma [1]. The standard method of treatment is a chemotherapy cocktail comprised of cyclophosphamide, hydroxydaunomycin, oncovin, and prednisone (CHOP), in combination with the monoclonal antibody rituximab. While some patients respond well to this treatment, 45–50% relapse, suggesting an improved treatment method is critical [2, 3]. Screening new therapies presents a major challenge, as currently available models fail to accurately recreate the DLBCL microenvironment. 2D in vitro models do not reflect the arrangement of DLBCL in a cancerous lymph node, and many cellular characteristics, such as morphology and metabolism, are different in 2D culture compared to their natural 3D environment [4]. Treatments that appear highly effective in vitro frequently do not translate to in vivo models or clinical success. Utilizing 3D in vitro models to better recreate the metastatic lymph node microenvironment is therefore logical to better screen potential treatments for DLBCL before dedicating resources to in vivo models.

Some attempts at creating a 3D in vitro model for culturing DLBCL have previously been made [5–8]. While these models have distinct improvements over 2D in vitro methods, many critical features of an optimal 3D model are missing. Most of these approaches lack a perfusion system to appropriately simulate a lymph node microenvironment [5–7]. Mannino et al. developed a model incorporating continuous perfusion, however, this approach lacked the capacity to directly monitor cell viability on-chip [8]. To create an improved platform for culturing and testing DLBCL, we applied our previously demonstrated droplet-based microfluidic spheroid model [9–12]. This device combines 1000 droplet docking sites with an alginate-based hydrogel to create aqueous-in-oil droplets laden with cells that can be cross-linked, continuously perfused, and imaged on-chip. This design is easy to fabricate and use and can be readily applied to test numerous treatment conditions in parallel. Critically, continuous perfusion allows biologically relevant delivery of nutrients and treatment conditions. In addition, this device allows for both real-time monitoring on-chip as well as downstream analysis through recollection of cells and continuous collection of secretions from the device’s array outlet. We utilized primary human DLBCL combined with primary NK cells, and treated these with Rituximab, CHOP chemotherapy, or the combination of both to make our model as clinically relevant as possible. Adding a cytotoxic immune cell to the model is necessary for a relevant response from the added treatments, as rCHOP treatment is primarily an immune-modulating therapy [13–15]. NK cells were chosen due to their ability to function independently of antigen-presenting cells, and because they are the most prominent immune cell in the DLBCL microenvironment [16]. We validated our model using various phenotypic and genotypic analytical methods to fully characterize the cellular response to treatments in 3D spheroids. The data we gathered reveal that DLBCL cells display greater resistance in our platform compared to 2D culture methods, better correlating to clinical expectations. This study demonstrates a method for rapidly and effectively screening novel therapeutic approaches, with potential novel observations of cellular response to rCHOP treatment.

## Materials and methods

Methods are described in expanded detail in the Supplementary Information.

### Cell culture

Diffuse large B-cells (DLBCL) were provided by the lab of Dr. Andrew Evens at Rutgers Cancer Institute, derived from metastatic lymph nodes isolated from patients. Cells were de-identified prior to delivery, and patient data was kept confidential from us. DLBCL were cultured in RPMI-1640 (ATCC, Manassas, VA) with 10% FBS and 1% Antibiotic/antimycotic mixture (Gibco, Waltham, MA). PB NK cells were purchased from Stemcell Technologies (Vancouver, BC, Canada). NK cells were thawed the day prior to experiments and rested overnight in RPMI-1640.

### Microfluidic device fabrication and filling

Devices were fabricated using standard soft lithography techniques with PDMS [9, 12]. Vitrogel-RGD High Concentration was purchased from TheWell Biosciences (North Brunswick, NJ). Alginate solution was either mixed with RGD Vitrogel or added alone to glass vials with a stir bar. Cells were pelleted and resuspended in HBSS, then added to alginate while continuously stirring. Alginate was made to 1% w/v final concentration, either without or supplemented with 7.5% v/v RGD+ Vitrogel. After ~30 s of stirring, the cell suspension was withdrawn into a 1-mL syringe, then perfused into our 3D devices with Tygon microbore tubing (Saint-Gobain Company, Courbevoie, France) using Harvard Apparatus syringe pumps (Holland, MA). Mineral oil with 2% v/v span-80 surfactant (MilliporeSigma, Munich, Germany) was utilized to create the aqueous-in-oil droplet emulsions. To crosslink the droplets, 125 mM calcium chloride solution in RPMI-1640 was perfused into the device.

### Cell preparation for imaging

DLBCL were incubated with 10 µM CMAC CellTracker (ThermoFisher) in serum-free media for 45 min and washed twice with media prior to pelleting and resuspending in hydrogel. NK cells were incubated with 2 µM CFSE CellTracker (Thermofisher) and resuspended with DLBCL for co-culture conditions. NK cells were loaded at a 1:2 ratio to DLBCL. NK cells were loaded at 5 to 7.5 million cells/mL, while DLBCL was loaded at 10 to 15 million cells/mL. For treated conditions, Rituximab was added to perfused media at 1 µM and CHOP was added at 250 ng/mL. Prior to viability imaging, devices were perfused with 8 µM ethidium homodimer (Biotium, Freemont, CA) for ~3 h. Spheroids were differentiated between low density and high density, with high density having >5 cells/mm^2^.

For immunofluorescent imaging, cells were labeled with fluorescently conjugated antibodies in droplets. For DLBCL, the antibodies used were anti-CD47 conjugated to PerCP-Cy5.5, anti-PDL1 conjugated to Alexa Fluor 594, and anti-CD20 conjugated to Brilliant Violet 421 (Biolegend). For NK cells, anti-SIRPα conjugated to FITC, anti-PD1 conjugated to Alexa Fluor 647, and anti-CD16 conjugated to PE were used for labeling (Biolegend). All images were taken at ×20 magnification.

## Results and discussion

### Hydrogel composition supports cellular biocompatibility

**Fig. 1:**
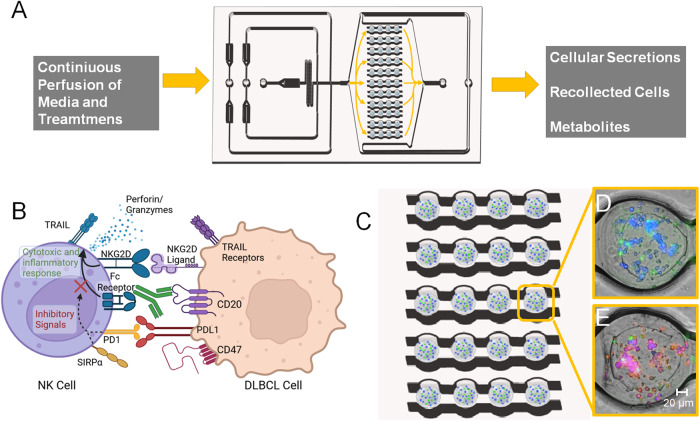
Overview of workflow and data output of the device. **A** Drawing of 3D device filled with cell-loaded hydrogel spheroids. Device allows continuous perfusion of nutrients and treatment conditions to spheroids, with simultaneous collection of secretions and metabolites. In addition, cells can be recollected after culture for downstream analysis. **B** Diagram depicting some of the biological characteristics observed in NK cells and DBLCL cultured in our spheroid platform. **C**, **D** Representation of images taken from spheroid devices. **C** Enlarged drawing of array loaded with hydrogel spheroids containing cell. **D**, **E** In all, 20× magnified images of spheroids containing DLBCL and NK cells. **D** Before rCHOP treatment and E) After rCHOP treatment. DLBCL are labeled in blue, NK cells are labeled in green, and ethidium homodimer labels dead cells with red fluorescence.

**Fig. 2:**
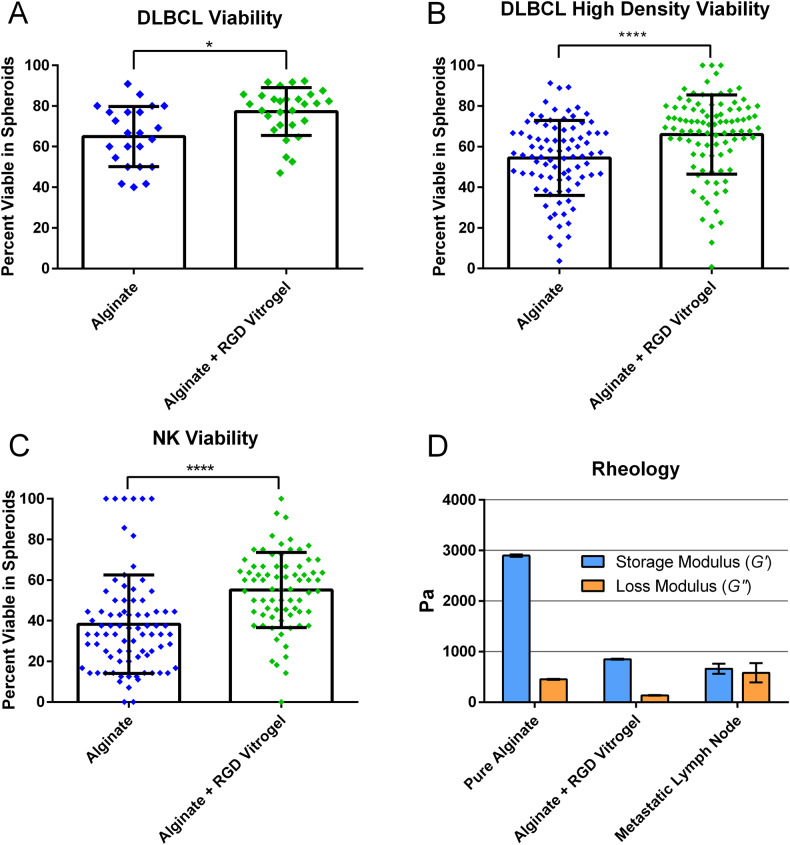
Characterization of alginate versus alginate supplemented with high concentration RGD vitrogel. **A**–**C** Comparison of viability in different hydrogel compositions after 24-h incubation in 3D device. **A** Comparison of DLBCL viability in low-density loaded spheroids (≤2 cells/mm^3^) after 24 h. *n*: Alginate = 20 spheroids, Alginate + RGD Vitrogel = 26 spheroids. **B** Comparison of DLBCL viability in high-density (>2 cells/mm^3^) spheroids. *n*: Alginate = 86 spheroids, Alginate + RGD Vitrogel = 93 spheroids. **C** NK viability comparison, *n*: Alginate = 82 spheroids, Alginate + RGD Vitrogel = 74 spheroids. **D** Storage modulus (G’) and loss modulus (G”) measurements of hydrogels compared to metastatic lymph node values found in literature.18 Viability statistical comparisons performed via unpaired *t* test. Rheology comparisons were performed via Tukey multiple-comparisons test. Error bars represent standard deviation. *P ¬< 0.05, ****P < 0.0001.

As depicted in Fig. 1, the 3D alginate spheroid platform allows for continuous culture of cells with sustained delivery of nutrients and treatment conditions to simulate circulation. Cell viability can be easily imaged in the array with fluorescent viability dyes (Fig. 1D, E). Alginate is a polysaccharide derived from brown algae that produces a biocompatible, permeable, and readily cross-linked hydrogel [17]. While biocompatible, alginate lacks attachment groups for cells to adhere, and can be highly rigid when cross-linked. To enhance DLBCL viability in the hydrogel, we supplemented alginate with an RGD-rich hydrogel (TheWell Biosciences). The RGD (arginine, glycine, and aspartic acid) enhances cell adhesion, which can promote enhanced viability in 3D cultures [17]. We used fluorescence microscopy to determine cell viability in our platform. We found this hydrogel formulation produced an increase in the average DLBCL viability after 24 h in low-density spheroids from 65% to 77% compared to alginate alone (Fig. 2A), while in high-density spheroids the viability increased from 54 to 66% (Fig. 2B). NK cells also displayed better viability in the spheroids with supplemented RGD^+^ hydrogel, increasing from 38 to 55% viability after 24 h (Fig. 2C). To determine whether our hydrogel had similar mechanical properties to a metastatic lymph node, we measured its storage modulus (stiffness/resistance to deformation) and loss modulus (the heat-energy dissipated), a viscoelastic property. Alginate with RGD Vitrogel displayed more similar rheological properties to those of metastatic lymph nodes found in literature, with overall mean difference of both metrics 1182.7 between pure alginate and lymph node and of 316.6 between alginate with RGD Vitrogel and lymph node [18]. While both differences are statistically significant based on two-way ANOVA with the Tukey test (*P* value < 0.01), alginate with RGD Vitrogel has notable closer stiffness (G’) to metastatic lymph nodes compared to pure alginate (Fig. 2D and Supplementary Table 1).

### 3D hydrogel culture promotes cell interaction and resistant phenotype

**Fig. 3:**
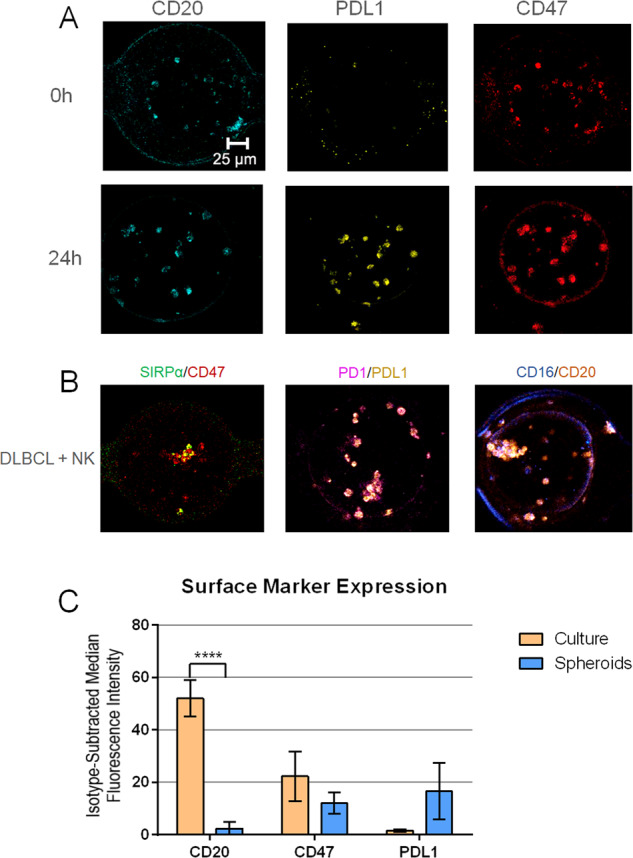
Characterization of surface marker expression in cells cultured in 3D spheroids. **A** Confocal microscopy images at ×20 magnification of DLBCL in 3D spheroids with surface markers labeled immediately after loading devices (0 h) and after 24 h of incubation. **B** Confocal microscopy images of DLBCL and NK co-culture in 3D spheroids after 24 h of incubation with labeling of interacting NK and DLBCL surface markers. SIRPα is labeled in green, CD47 in red, PD1 in magenta, PDL1 in yellow, CD16 in blue and CD20 in orange fluorescence. Antibodies and associated fluorophores are described in detail in Supplemental Methods. **C** Flow cytometry surface marker analysis of DLBCL either recollected from 3D after 24 h of incubation or taken directly from suspension culture. *N* = 3 experimental repeats for each condition, statistical significance determined by Tukey’s multiple-comparisons test. ****P value < 0.0001.

Next, we utilized confocal microscopy to observe the expression of certain surface markers associated with the prognosis of clinical DLBCL cases. DLBCL were labeled with CD47 and PDL1 monoclonal antibodies, two checkpoint inhibitor ligands associated with resistance to immunity, and for CD20, the target of rituximab [16, 19–21]. NK cells were labeled for SIRP1α and PD1, the receptors for CD47 and PDL1, respectively, and for CD16, a key factor in antibody-dependent cellular cytotoxicity [22]. Cells were labeled and fixed on-chip, then observed through confocal microscopy. To see changes in expression levels over time, we loaded devices with DLBCL and labeled cells immediately after loading, and compared those to devices incubated for 24 h (Fig. 3A). We observed a slight visual increase in expression in CD20 and CD47 from time 0 to 24 h. Interestingly, we saw no substantial labeling of PDL1 at 0 h, but notable expression at 24 h. This indicates a resistant phenotype developing over time in the 3D hydrogel environment.

To observe the interaction between DLBCL and NK, we loaded devices with both cell types, incubated them for 24 h, and observed the labeling of surface markers that interact on each cell type (Fig. 3B). Based on the localization of the fluorescent signal, we see clear clustering of NK and DLBCL in the spheroids, indicating the interaction of the two cell types. This provides qualitative evidence that NK cells and DLBCL are interacting in the spheroids, allowing accurate modeling of lymphocyte infiltration of a metastatic lymph node.

To quantifiably observe differences between surface expression of DLBCL in our 3D platform compared to those taken directly from culture, we next tested the cells via flow cytometry. Monoclonal antibodies targeting PDL1, CD47, and CD20 were compared to a non-specific isotype control to determine degree of expression. All antibodies possessed the same fluorophore (PerCP Cy5.5) to improve direct comparison of expression levels. DLBCL were loaded onto 3D devices following our standard protocol and incubated for 24 h. To prevent loss of surface marker expression, DLBCL were labeled and fixed on-chip before recollecting cells. DLBCL taken directly from culture were labeled with the same method, then analyzed with flow cytometry. We found significantly higher levels of CD20 were expressed on the DLBCL taken directly from culture than those from 3D (Fig. 3C). In addition, though not statistically significant, a notable increase in median PDL1 expression was observed in cells collected from 3D. CD47 displayed a minor, statistically insignificant decrease in the 3D condition.

### DLBCL response to treatments in spheroids matches expectations of resistant phenotype

**Fig. 4:**
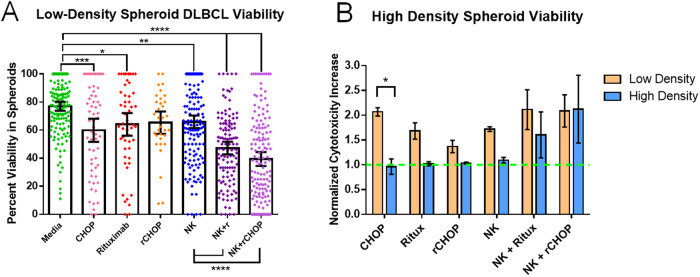
Analysis of DLBCL survival in 3D alginate + RGD Vitrogel spheroids after 24-h incubation. **A** Viability of DLBCL in spheroids loaded at ≤2 cells/mm^3^. N: Media = 150, CHOP = 59, Rituximab = 54, NK = 149, NK+r = 120 and NK+rCHOP = 150 spheroids. **B** Comparison of DLBCL survival across treatment conditions in low-density versus high-density spheroids, normalized to media-only control. *N* of high-density experiments: Media = 72, CHOP = 89, Rituximab = 72, rCHOP = 88, NK = 35, NK+r = 46 and NK+rCHOP = 37 spheroids. Statistical analysis based on one-way ANOVA with Tukey multiple-comparisons test. Error bars represent 95% confidence interval of mean. *P ¬<0.05, **P < 0.01, ***P < 0.001 ****P < 0.0001.

To see how DLBCL responded to standard chemotherapy in our 3D model, we loaded devices with DLBCL cells with and without NK cells, and perfused the devices with various treatment conditions for 24 h to simulate relevant in vivo treatment. We observed modest but significant decreases in DLBCL viability using each treatment condition alone. Interestingly, the combination of Rituximab and CHOP did not increase DLBCL killing in the absence of NK cells. In the combination conditions (NK + Rituximab and NK + rCHOP), we found an additional significant decrease in viable DLBCL cells (Fig. 4A). The combination of NK + rCHOP had the greatest overall decrease in DLBCL viability, from approximately 75% to 40% viable cells in droplets after 24-hour treatment. To see how increasing cell density affects DLBCL susceptibility to killing, we repeated the same series of conditions with our high-density loaded spheroids. We found that the ability of CHOP alone to kill DLBCL was significantly reduced (Fig. 4B). All other conditions resulted in a non-significant decrease in DLBCL killing except for the NK + rCHOP combination, which produced a similar increase in cytotoxicity.

For a direct comparison to a traditional 2D in vitro model, we repeated the treatment conditions using a standard colorimetric LDH-release assay in a 96-well plate, loading DLBCL and NK at concentrations relevant to the 3D spheroids, and incubating for 24 h. We found even less killing of DLBCL with this assay, however, trends were similar to our 3D data (Supplementary Fig. 1A). While the combination of all treatments remained the most effective, rituximab and CHOP alone or in combination produced more cytotoxicity than NK alone or NK + Rituximab. For additional comparison, we applied calcein AM to observe cell viability directly in plates. While trends across treatments closely matched the LDH-release assay, a higher percent killing was indicated for all conditions (Supplementary Fig. 1B).

### Cellular secretions vary across treatment conditions

**Fig. 5:**
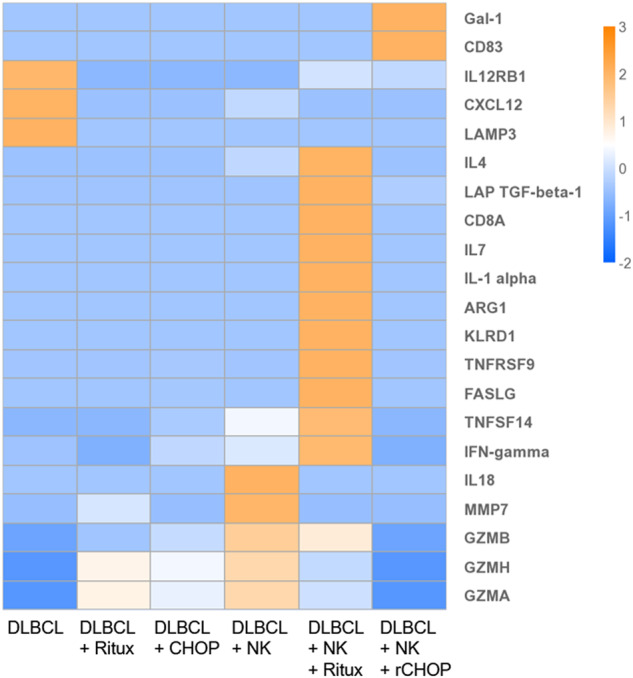
Analysis of normalized, comparative expression levels of secretions found in perfusions collected from 3D spheroids over 24-h incubation. Data represents mean NPX values of two replicates, and all results are adjusted to cell concentration in devices. Values are then scaled with log2 transformation for visual comparison via heatmap.

While devices undergo overnight perfusion, the flow-through was collected from individual treatment conditions to analyze secretions released from the DLBCL and NK. For correlation to our viability data, perfusions were continuously collected from the device during the first 24 h of culture. Of the 96 markers analyzed, 21 were found in our collected samples of interest (Fig. 5). In the secretions of DLBCL alone, lysosome-associated membrane glycoprotein 3 (LAMP3), C-X-C motif chemokine 12 (CXCL12) and IL12 receptor (IL12RB1) were found at concentrations threefold higher than most other samples. DLBCL with Rituximab alone and with CHOP alone displayed similar secretion levels, with no proteins found at notably elevated levels compared to other conditions. The condition with DLBCL and NK displayed the highest levels of granzyme (GZM) secretions, which were reduced with the addition of treatments. Interestingly, some levels of Granzyme H and Granzyme A were observed in DLBCL with Rituximab, which have not been previously reported in DLBCL. DLBCL + NK + Rituximab showed expression approximately threefold higher than other samples of numerous alternative factors associated with immune cell activation and inflammation, including IL4, IL7, IL1α, and IFNγ, alongside several cell surface markers present in active cells [23–32]. These markers are all reduced with the addition of CHOP in the DLBCL + NK + rCHOP condition. Instead, Gal-1 and CD83 are found at increased levels. Overall, analysis of proteins collected in the device flow-through demonstrates a highly variable cellular microenvironment across treatment conditions.

### Transcriptomic sequencing reveals mechanisms driving treatment efficacy

To further characterize the difference in cellular characteristics between treatment conditions in this platform, we decided to perform a transcriptomic analysis on cells in the DLBCL + NK + Rituximab and the DLBCL + NK + rCHOP conditions for comparison. To match viability observations, cells were collected after 24 h of culture. Alginate was dissolved with a chelating solution, and cells were collected. To avoid changes in transcriptomic profiles and loss of integrity, cells were immediately lysed after collection, and cellular RNA was extracted.

**Fig. 6:**
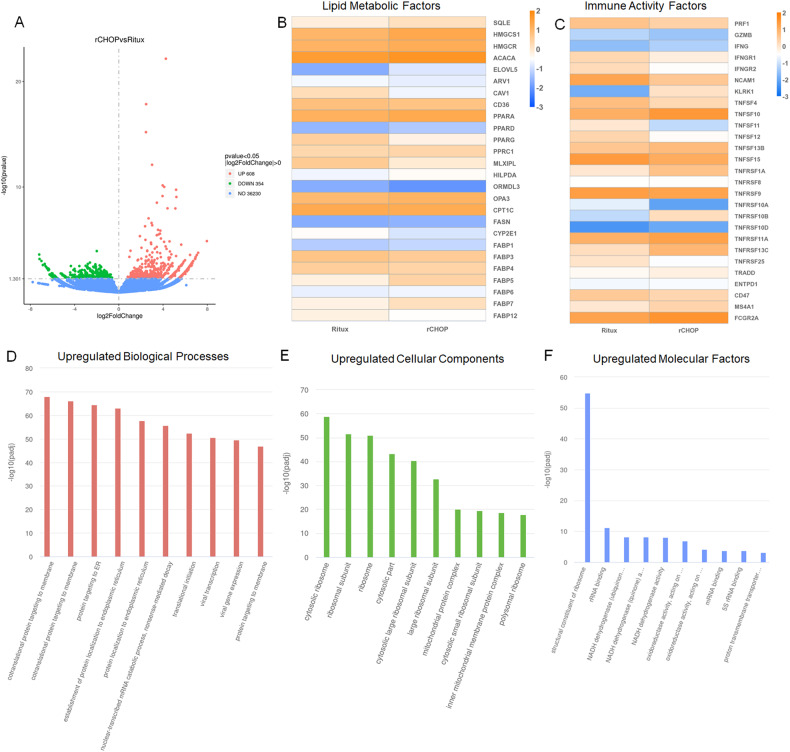
Transcriptomic sequencing data of DLBCL and NK recollected from 3D devices after 24 h of incubation, treated either with rCHOP or Rituximab alone. **A** Volcano plot demonstrating the total quantity of genes upregulated, downregulated, and unchanged between rCHOP and Rituximab-treated cells. **B** Heatmap demonstrating log2 scaled fold change in read counts between treatments, focusing on genes associated with lipid production and metabolism. **C** Heatmap demonstrating the log2 change in read counts between conditions, focusing on genes associated with NK cell activity. **D**–**F** Gene Ontology (GO) data showing pathways with the highest upregulated genes in rCHOP-treated cells, broken up by **D** Biological Processes, **E** Cellular Components and **F** Molecular Factors.

Looking at the overall gene expression comparison of these samples, we see 608 genes are upregulated and 354 are downregulated in the rCHOP treatment compared to Rituximab alone (Fig. 6A). When looking directly at the expression of the top 30 most differentially expressed genes, mitochondrial, ribosomal, and structural genes appear to be the most variable between populations (Supplementary Fig. 2). Mitochondrial NADH expression trended higher in Rituximab-treated cells, while Mitochondrial RNA related genes were higher in rCHOP treated. Other gene families, such as mitochondrial cytochromes, and structural genes, were variable, with some genes elevated in each condition. Reviewing the Gene Ontology (GO) analysis of these samples, ribosome-related genes are revealed to be highly expressed in rCHOP, both in Cellular Components and in Molecular Processes (Fig. 6E, F). This upregulation insinuates more bioactivity in the rCHOP sample, suggesting potential metabolic and activity shifts in response to CHOP. Looking at expression of the top-expressed ribosomal proteins and ribosome biogenesis genes, we see a mixed response for each treatment (Supplementary Fig. 3). Across ribosomal proteins, an average increase in expression is found in rCHOP, except mitochondrial ribosomal proteins, which were found to be slightly higher in Rituximab alone treatment. In addition to ribosome upregulation, protein trafficking and enzymatic activity, such as NADPH and oxidoreductase expression, were found to be upregulated in rCHOP-treated cells (Fig. 6D–F).

To investigate the metabolic activity differences between these conditions, we focused on the expression of genes related to lipid and fatty acid synthesis and metabolism (Fig. 6B). CAV1, CD36, MLXIPL, CPT1C, and CYP2E1 were highly expressed in the cells treated with Rituximab alone. Increased lipid metabolic activity has been correlated with cancer resistance, and these markers have specifically been shown to be upregulated in cancers with poor prognosis [33, 34]. Several lipid factors were also upregulated in the rCHOP conditions, including ACACA, HMGCs, FASN, and SQLE. We also looked at several fatty acid binding proteins (FABPs) and Peroxisome Activator Receptors (PPARs). FABP5, 7 and 12, and PPARA and D were found at higher levels in rCHOP-treated cells. FABP and PPAR expression has been found to correlate to NK cells maintaining critical cytotoxic capabilities in the lymphoma microenvironment [35]. FABPs and PPARs also can contribute to DLBCL resistance, however, and their upregulation may be another sign of these cells trying to resist treatment [36, 37].

We also pulled out the expression of several major factors in immune activity and cytotoxicity (Fig. 6C). While little difference was observed in perforin, granzyme, and IFNγ expression, expression levels varied considerably amongst the TNF family of receptors and ligands. Of particular interest, TNFSF10 (TRAIL) was upregulated, while TNFSF11 (RANKL) and TNFSF12 (TWEAK) were downregulated. TRAIL ligand is expressed on NK and other immune cells and can directly induce apoptosis in target cells [38, 39]. In addition, TRAIL has previously been shown to drive NK-mediated cytotoxicity in treatment with doxorubicin, one of the components of CHOP [40]. RANK and TWEAK pathways, conversely, have been shown to promote cancer cell survival, suggesting they may be providing resistance to the DLBCL which is reduced with CHOP treatments [38]. High expression of TNFRSF10B, a TRAIL receptor that induces apoptosis, was also observed in the rCHOP condition [41]. NCAM1 (CD56) was higher in Ritux alone, while FCGR2A, one of the components of the NK Fc receptor, was higher in rCHOP. This may relate to higher expression of CD16 in the rCHOP condition, which tends to be inversely correlated to CD56 expression. In addition, the expression of KLRK1 (NKG2D) was found to be much higher in the rCHOP condition. NKG2D receptor binding incites cytokine production and cytotoxicity in NK cells, however, overstimulation can lead to tolerance induction [42].

## Discussion

To establish our model as a suitable approach for DLBCL treatment screening, we first tested hydrogel compatibility with cells. While device loading and hydrogel crosslinking produced a sharp initial decrease in viability, this is a common occurrence in 3D hydrogels [43–45]. In addition, adequate cell viability remained to observe expected differences between treatment conditions, as seen in Fig. 4. The cell viability and rheological characteristics of this hydrogel formulation were deemed adequate for continued characterization of DLBCL response to this culture system. We next assessed the development of a biologically relevant microenvironment through testing of key surface marker expression levels and comparing these to cells taken from suspension culture. Confocal microscopy and flow cytometry confirmed a resistant phenotype in 3D culture, with reduced chance of rituximab effect and potential for PDL1-mediated resistance in some cells.

To determine whether response to treatment matched in vivo expectations, we tested the rCHOP treatment in our device (Fig. 4). Compared to plate data (Supplementary Fig. 1), we observed a response to treatment far closer to biological relevance. Responses to treatment conditions in spheroids matched findings from in vivo models, where individual treatments can directly kill DLBCL, however the direct activity of CHOP combined with the immune modulation of rituximab work together to drastically enhance cytotoxicity [15, 46–49]. As expected, some DLBCL resisted treatment in all conditions, and resistance increased with higher DLBCL densities. NK-mediated cytotoxicity was reduced in plates compared to 3D, potentially due to reduced ability for NK cells to interact directly with DLBCL in solution compared to in a hydrogel. Rituximab and CHOP had much higher efficacy in plates than expected, likely due to increased direct exposure to DLBCL. In clinical studies, rCHOP has been shown to have much higher rates of patients with complete responses than patients treated with Rituximab alone [50]. The level of killing observed with Rituximab alone in the plate assays is much higher than should be expected based on this clinical data. These results may be influenced by the drastically reduced expression of CD20 in spheroids compared to expression seen in DLBCL cells taken directly from culture, as observed in Fig. 3C. Ultimately, these findings suggest a traditional plate assay produces data that contradicts clinical responses and is less reproducible than our 3D model.

To further enhance our understanding of cellular behavior in the spheroids, we measured changes in cellular secretions and transcriptomics in response to the different treatment conditions. Secretion analysis revealed several unexpected differences between treatment conditions. In DLBCL alone, lysosome-associated membrane-protein 3 (LAMP3), a marker associated with metastasis from lymph nodes, was found threefold higher than in other conditions [51]. Other factors expressed in this condition are CXCL12 and IL12 receptor (IL12RB1), both factors known to help activate cytotoxic immune cells [52, 53]. The NK + Rituximab treatment possessed increased levels of various proteins associated with inflammation. This result is logical, as rituximab binding activates the ADCC pathway in NK cells which should produce a highly active and cytotoxic phenotype [14, 54]. Several of these secretions, however, also have been shown to promote immune cell evasion in late-stage cancers, and can lead to immune cell desensitization or exhaustion [26, 30, 32, 55–57]. Interestingly, we found that the addition of CHOP did not reflect the same inflammatory profile, with only levels of Gal-1 and CD83 found at elevated levels. Gal-1 has been correlated to increased metastasis and immune evasion, while CD83 is known to decrease NK cell activation [58, 59]. These changes may be influenced by the anti-inflammatory activity of prednisone in the CHOP [13]. This data is collected from a single timepoint, however, and may be heavily influenced by cell cycle. To add additional understanding to the factors affecting cellular phenotype between the Rituximab alone and rCHOP-treated DLBCL and NK, we recollected cells for transcriptomic sequencing at the same 24-h timepoint.

Our transcriptomic data revealed signs of higher metabolic activity in rCHOP treatment compared to Rituximab alone. Given that mRNA in these samples would be isolated from cells that survived treatment, these might suggest pathways involved in surviving the different treatment conditions. The most significant Gene Ontology (GO) terms that came up for these two samples included ribosome and enzyme expression, suggesting metabolic differences (Fig. 6D, E) [60–62]. In rCHOP, both ribosome and lipid metabolic factors were elevated (Fig. 6B and Supplementary Fig. 3). Ribosome RNA expression has been shown to have a range of consequences in cancer and other diseases, correlating to both cancer resistance and NK cell activity [62–64]. Mitochondrial ribosome proteins were higher in Rituximab, which may also be linked to resistance [65]. Several of the lipid factors found elevated in the rCHOP-treated cells have been correlated to lymphocyte activation, and it may be possible that they are signs of enhanced NK metabolic activity, however, enhanced lipid metabolisms has also been linked to impaired NK cell function [35, 61]. These data suggest higher metabolic activity is need for DLBCL to survive the addition of CHOP. Metabolic changes in response to external stress has been well-documented in cancer cells [60]. These metabolic changes could contribute to NK cell recognition of DLBCL.

Based on our combined secretomic and viability data, NK cells are able to maintain cytotoxicity despite an observed reduction in secretions in the rCHOP condition compared to Rituximab alone. Based on our transcriptomic sequencing data, metabolic changes in DLBCL required to survive the added CHOP cocktail may make them easier to recognize by NK cells. This is supported by CD20 expression, which was found higher in rCHOP-treated cells (log2 expression of 5.2 as opposed to 4.2 in Rituximab alone). In addition, chemotherapies for cancer are well-documented to promote tumor antigen presentation and incite immune cell cytoxicity [66]. Increased levels of NKG2D and TRAIL in the rCHOP condition support the potential of contact-based cytoxicity driving the DLBCL killing in this condition. Alternatively, the stress induced by CHOP administration may make DLBCL easier to eliminate through standard immune effector function.

Combining our results, we concluded that this model provides a powerful tool for testing the efficacy of lymphoma treatments. Using this platform, novel immunotherapies can be screened and directly compared to the current standard of care with greatly enhanced reliability compared to standard 2D methods. In addition, biological insights may help thoroughly characterize an expected in vivo cellular response to treatment, which can contribute to more careful tailoring of treatment design. Given the extensive variety of novel therapies and combinatorial approaches for treating lymphoma, enhanced models for early screening are essential to expedite and improve the quality of early drug development.

### Reporting summary

Further information on research design is available in the Nature Portfolio Reporting Summary linked to this article.

### Supplementary information

Supplemental Material

Reporting Summary Checklist

## Data Availability

Transcriptomic sequencing data is available on the NIH’s GEO database, under accession number GSE240989. All other data are available upon request.
