# Supplementary material for: Characterizing influence of rCHOP treatment on diffuse large B-cell lymphoma microenvironment through in vitro microfluidic spheroid model
Source: Cell Death Dis. 2024 Jan 9;15(1):18. doi: 10.1038/s41419-023-06299-6 (PMC10776622; doi:10.1038/s41419-023-06299-6)
Supplement: Supplementary file 1 — Supplemental Material [file 41419_2023_6299_MOESM1_ESM.docx]

**SUPPLEMENTAL METHODS**

**Microfluidic Device Fabrication**

Devices were fabricated using standard soft lithography techniques with PDMS.^[8, 11]^ Silicon wafers were spincoated with SU-8 2075, and UV crosslinked in two stages, one to make the perfusion channels and one to make the device design. Channel heights were made to approximately 120 µm, with perfusion channels an additional 40 µm. Devices were made with 10:1 PDMS and curing agent and bonded to glass slides with a Harrick Plasma Cleaner (Harrick Plasma Inc, Ithaca, NY). Devices were made hydrophobic by perfusing aquapel (PPG Industries, Inc, Pittsburgh, PE) through the channels and allowed to evaporate after filling.

**Cell Culture**

Diffuse large B-cells (DLBCL) were provided by the lab of Dr. Andrew Evans at Rutgers Cancer Institute, derived from metastatic lymph nodes isolated from patients. Cells were de-identified prior to delivery, and patient data was kept confidential from us. DLBCL were cultured in RPMI-1640 (ATCC, Manassas, VA) with 10% FBS and 1% Antibiotic/antimycotic mixture (Gibco, Waltham, MA). PB NK cells were purchased from Stemcell Technologies (Vancouver, BC, Canada). NK cells were thawed the day prior to experiments and rested overnight in RPMI 1640.

**Device Filling and Hydrogel Preparation**

Alginate provided to our lab by Dr. Smadar Cohen at Ben-Gurion University of the Negev. Alginate stocks were prepared at 4% in distilled water and filtered with a 0.22 µm filter. Vitrogel-RGD High Concentration was purchased from TheWell Biosciences (North Brunswick, NJ). Alginate solution was either mixed with RGD Vitrogel or added alone to glass vials with a stir bar. Cells were pelleted and resuspended in HBSS, then added to alginate while continuously stirring. Alginate was made to 1% w/v final concentration, either without or supplemented with 7.5% v/v RGD+ Vitrogel. After approximately 30 seconds of stirring, the cell suspension was withdrawn into a 1 mL syringe, then perfused into our 3D devices with Tygon microbore tubing (Saint-Gobain Company, Courbevoie, France) using Harvard Apparatus syringe pumps (Holland, MA). Mineral oil with 2% v/v span-80 surfactant (MilliporeSigma, Munich, Germany) was utilized to create the aqueous-in-oil droplet emulsions. Once the docking sites of the droplet array were satisfactorily filled, the perfusion of cells and oil was stopped. To crosslink the droplets, 125 mM calcium chloride solution in RPMI 1640 was perfused into the device at a low flow rate, approximately 15 to 50 µL/h. Once the calcium solution displaced all oil from the device, the syringes were replaced with complete media, including the relevant treatment conditions, and perfusion was maintained at 50 µL/h for the remainder of the experiment.

**Viability Imaging and Cell Preparation**

For viability imaging, an Axio Observer equipped with an automated stage and incubation chamber for maintaining cells at 37 °C and 5% CO_2_. The microscope is also equipped with a fluorescent light source and filter set for DAPI, EGFP, dsRed and far-red channels. DLBCL were incubated with 10 µM CMAC CellTracker (ThermoFisher) in serum-free media for 45 minutes and washed twice with media prior to pelleting and resuspending in hydrogel. NK cells were incubated with 2 µM CFSE CellTracker (Thermofisher) and resuspended with DLBCL for co-culture conditions. NK cells were loaded at a 1:2 ratio to DLBCL. NK cells were loaded at 5 to 7.5 million cells/mL, while DLBCL were loaded at 10 to 15 million cells/mL. For treated conditions, Rituximab was added to perfused media at 1 µM and CHOP was added at 250 ng/mL. Prior to viability imaging, devices were perfused with 8 µM ethidium homodimer (Biotium, Freemont, CA) for approximately 3 hours. Both CMAC and CFSE CellTrackers remain present after cell death, allowing differentiation of live and dead DLBCL and NK based on the CellTracker fluorescence, and the presence or absence of red fluorescence. Spheroids were differentiated between low density and high density, with high density having >5 cells/mm^2^.

**Immunofluorescent Imaging**

For immunofluorescence imaging, a Zeiss LSM 880 confocal microscope was used (Zeiss). This microscope was equipped with 405, 458, 488, 514, 561, 594 and 640 nm excitation lasers, Airyscan super-resolution and spectral unmixing capabilities. For this imaging, cells were not labeled with dyes prior to loading spheroids. All labeling antibodies utilized were mouse monoclonal IgG acquired from Biolegend (San Diego, CA). For DLBCL, the antibodies used were anti-CD47 conjugated to PerCP-Cy 5.5 (Cat. No. 323109, Clone CC2C6), anti-PDL1 conjugated to Alexa Fluor 594 (Cat. No. 329742, Clone 29E.2A3), and anti CD20 conjugated to Brilliant Violet 421 (Cat. No. 302329, Clone 2H7). For NK cells, anti-SIRPα conjugated to FITC (Cat. No. 372107, Clone 15-414), anti-PD1 conjugated to Alexa Fluor 647 (Cat. No. 367419, Clone NAT105), and anti-CD16 conjugated to PE (Cat. No. 302007, Clone 3G8) were used for labeling. Prior to imaging, devices were infused with antibodies in serum-free RPMI-1640 at 50 µL/h for 2 hours. After antibody labeling, cells were fixed by perfusion BD fixation buffer () for 1 hour, then washed with HBSS via perfusion for 1 hour). Images were done at 20x magnification. Unlabeled spheroids loaded with an identical cell concentration were used as controls for autofluorescence.

**Plate Cytotoxicity Assay**

To match the concentrations in spheroids, 96 well plates were loaded with 25,000 DLBCL per well, and 12,500 NK cells per well in 100 µL of media and incubated for 24 hours. As with the 3D experiments, Rituximab was loaded at 1 µM and CHOP was loaded at 250 ng/mL for relevant treatment wells. Wells with NK cells without DLBCL were included as a control for all the co-culture treatment conditions. Positive control wells were lysis with the 10X lysis solution included in the Promega Cytotox 96 Non-radioactive plate kit. After 24h incubation, supernatant was collected and LDH release was observed according to the assay protocol, using a ThermoFisher Varioskan Lux plate reader for absorbance readings. Additionally, cell calcein AM (Invitrogen) and ethidium homodimer III (Biotium, Freemont, CA) was added to the plate at 2 µM each. Fluorescence was measured using the Varioskan plate reader. Graphed values represent the NK + Treatment wells subtracted from the NK + DLBCL + Treatment wells, and divided by the lysed positive control wells, as represented in the below equation.

$$\frac{Effector+Target Signal-Effector Spontaneous Signal}{Target Max Signal}$$

**Rheology**

Rheological measurements of hydrogels were measured using a TA Instruments Discovery HR-1 Hybrid Rheometer with 40 mm cone and plate geometry and 1.007° cone angle (TA Instruments, New Castle, DE). Hydrogels were prepared by filling a 40 mm glass petri dish with a layer of alginate or alginate/Vitrogel mixture, and crosslinked with an equal volume of 250 mM calcium chloride solution in media. Storage and loss modulus were measured at 37°C, with a frequency of 10 rad/s, and a constant 2% strain. Graphed values represent average measurements from every 10 seconds over a 70 second run.

**Transcriptomic Sequencing**

For transcriptomic sequencing, cells were first recollected from hydrogels. To recollect cells, a 10 mM sodium citrate solution (MilliporeSigma) supplemented with 50 mM EDTA (Gibco) was perfused through devices at 200 µL/h. Devices were kept on the microscope at 37 °C and monitored constantly. Perfusion was performed for approximately 30 – 45 minutes, until alginate was completely un-crosslinked. Total RNA was extracted from collected cells using the RNeasy Mini Kit (QIAGEN, Venlo, Netherlands), then frozen at -80 °C. Frozen samples were sent to Novogene (Beijing, China) for transcriptomic analysis utilizing Takara SMART-seq V4 kit for amplification and the Illumina NovaSeq platform for sequencing. Data processing and bioinformatics is provided by Novogene. Two replicates were sequenced for each tested condition. Heatmaps were generated with Novogene’s NovoMagic tool. This software scales normalized read counts by sample, setting the mean read count as 0 and displaying the log2 fold differences between genes. Statistical significance calculated by Novogene, using p value and multiple hypotheses testing to create adjusted p values.

**Flow Cytometry**

To label cells for flow cytometry, mouse monoclonal antibodies conjugated to PerCP-Cy5.5 were used (Biolegend). Catalog numbers 329737 (Clone 29E.2A3), 621613 (Clone A17188B), 30325 (Clone CC2C6), 323109 (Clone CC2C6) and 400338 (clone MPC-11) were used to label PD-L1, PD-1, CD20, CD47 and Isotype Control, respectively. Separate devices were used for each antibody labelling. Devices were labeled and fixed as previously described for the immunofluorescence imaging Experimental Section 4.5). After washing, cells were recollected as previously described for transcriptomic sequencing (Experimental Section 4.8). For cells from culture, cells were collected into separate samples for each antibody at approximately 400,000 cells per vial, and labeled with 5 µL antibody in 200 µL BD FACS Staining Buffer (Becton, Dickinson and Company, Franklin Lakes, New Jersey) for 30 minutes at 4 °C. After labeling, cells were washed then fixed with BD Fixation Buffer and washed one more time with Staining Buffer. Cells were analyzed using a BD FACSCalibur with a 488 nm laser and 585/42 filter. Results were normalized to isotype control.

**Secretion Analysis**

During overnight perfusion of 3D spheroid devices, media flow-through was collected for some experiments. Perfused media was collected in separate 2 mL microcentrifuge tubes and stored at -80°C. Samples were later randomized and plated, then sent to Olink Proteomics (Waltham, MA) frozen on dry ice for analysis of secretions. Samples were tested using the Olink Target 96 Immunooncology panel, which tests for 96 proteins and peptides of interest related to immune and cancer cell interactions. The Proximity Extension Assay (PEA) technology used for the Olink protocol has been well described,^[65]^ and enables 96 analytes to be analyzed simultaneously, using 1 µL of each sample. The final assay read-out is presented in Normalized Protein eXpression (NPX) values, which is an arbitrary unit on a log2-scale where a high value corresponds to a higher protein expression. The internal controls are designed to mimic and monitor the different steps of the PEA. They consist of two incubation/immuno controls, an extension control, and a detection control. The internal controls are introduced to all samples as well as to the external controls and are used for quality control and normalization of the data. The external controls consist of a negative control used to calculate the limit of detection (LOD), as well as a triplicate of interplate controls (IPCs) that are used for data normalization. Quality control of the data is performed in two steps: First, the run is quality controlled by calculating the standard deviation for the detection control and the incubation/immuno controls. The standard deviation should be below 0.2 for a run to pass quality control. Secondly, each sample is quality controlled by comparing the results for the detection control and one of the incubation controls against the run median. Samples that fall more than 0.3 NPX from the run median with regards to these two internal controls will fail the quality control.

**SUPPLEMENTAL DATA**


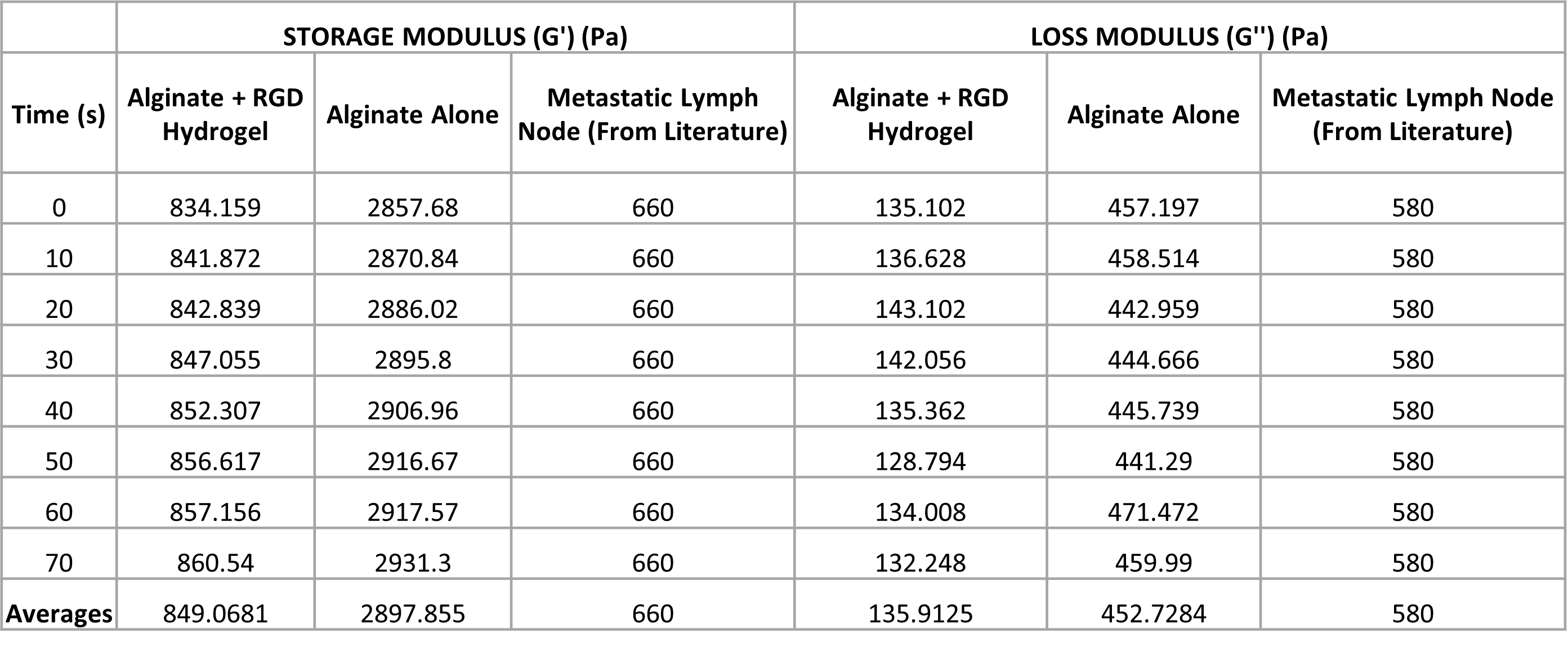


**Supplemental Table 1.** Measured rheological values of Alginate alone and Alginate + RGD Vitrogel compared to metastatic lymph node rheological data found in literature. Measurements made every 10 seconds for 8 total timepoints, and averages compared for statistical analysis.


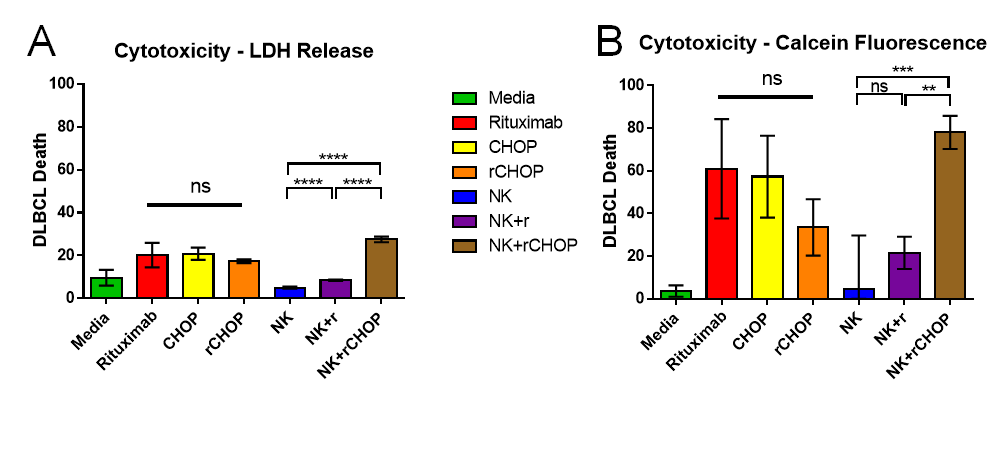


**Supplemental Figure 1.** Plate assay measuring effect of various treatments on DLBCL viability. A) Viability measured via LDH release via Promega Non-Radioactive assay. B) Viability measured by directly labeling cells with calcein green at end of incubation. Y axis represents approximate percent of DLBCL surviving at end of 24h incubation. Statistical significance determined by one-way ANOVA.


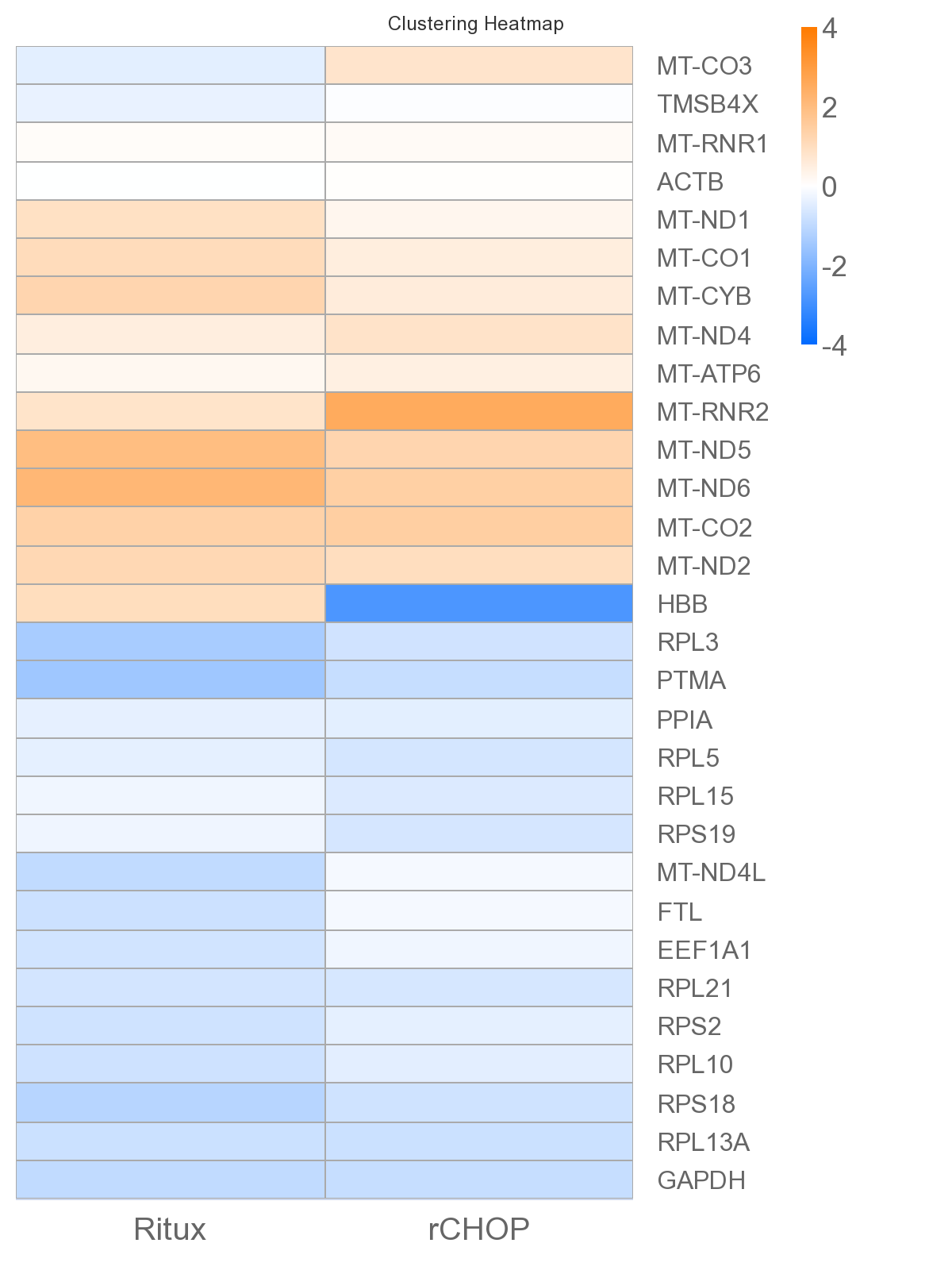


**Supplemental Figure 2.** Heatmap representing the log2 scaled read counts of the 30 most differentially expressed genes between Rituximab and rCHOP treatments.


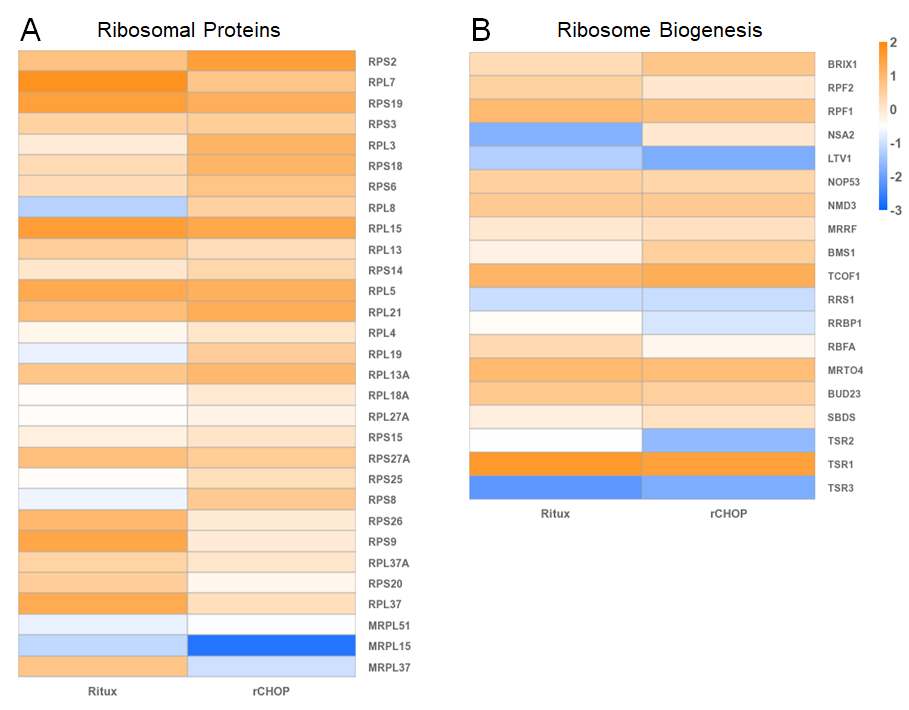


**Supplemental Figure 3.** Heatmaps of log2 scaled mRNA read count averages between Rituximab and rCHOP, separated by A) Ribosomal proteins and B) Ribosome biogenesis factors. Values represent average of two experimental replicates
